# Biocompatibility Characteristics of Titanium Coated with Multi Walled Carbon Nanotubes—Hydroxyapatite Nanocomposites

**DOI:** 10.3390/ma12020224

**Published:** 2019-01-10

**Authors:** Jung-Eun Park, Yong-Seok Jang, Tae-Sung Bae, Min-Ho Lee

**Affiliations:** Department of Dental Biomaterials and Institute of Biodegradable material, Institute of Oral Bioscience and BK21 plus project, School of Dentistry, Chonbuk National University, Jeonju 54896, Korea; pje312@naver.com (J.-E.P.); yjang@jbnu.ac.kr (Y.-S.J.); bts@jbnu.ac.kr (T.-S.B.)

**Keywords:** carbon nanotube, hydroxyapatite, nanocomposites, biocompatibility, titanium

## Abstract

Multi walled carbon nanotubes-hydroxyapatite (MWCNTs-HA) with various contents of MWCNTs was synthesized using the sol-gel method. MWCNTs-HA composites were characterized by X-ray diffraction (XRD) and transmission electron microscopy (TEM). HA particles were generated on the surface of MWCNT. Produced MWCNTs-HA nanocomposites were coated on pure titanium (PT). Characteristic of the titanium coated MWCNTs-HA was evaluated by field-emission scanning electron microscopy (FE-SEM) and XRD. The results show that the titanium surface was covered with MWCNTs-HA nanoparticles and MWCNTs help form the crystalized hydroxyapatite. Furthermore, the MWCNTs-HA coated titanium was investigated for in vitro cellular responses. Cell proliferation and differentiation were improved on the surface of MWCNT-HA coated titanium.

## 1. Introduction

Treatment for bone loss resulting from bone disease or bone defects caused by an accident typically requires a bone transplant [1]. Hydroxyapatite ((Ca_10_(PO_4_)_6_(OH)_2_; (HA)) is a potassium phosphate-based bioceramic material and is the main bone substitute used for such treatment. Its chemical components are similar to that of human bone or teeth and have excellent biocompatibility and bioactivity. As HA exhibits osteoconduction, which promotes osteoblast activities, and osteoinduction, which induces cytodifferentiation, it is widely used as a bone alternative or a composite material in dentistry and orthopedics [2,3]. However, the mechanical properties, such as its strength and fracture toughness, are weak due to the highly brittle nature of ceramic HA. Hence, it cannot be used alone in areas of the body that bear substantial weight. However, ceramic HA can be easily applied by coating it on the surface of metal biomaterials, such as titanium, cobalt-chromium alloys, or stainless steel. In such cases, the outstanding mechanical properties of metal and the excellent biocompatibility of ceramics are provided simultaneously [4,5]. To create the mechanical properties in an HA coating layer, many studies have reported adding a different substance as a reinforcing agent to HA, such as partially stabilized zirconia [6], or alumina [7]. However, a considerable amount of reinforcing agent is required to yield the desired mechanical properties, and existing reinforcing agents are either inactive in the body or have lower bioactivity than HA. HA complexes combined with a large volume of reinforcing agents exhibit reduced coherence with bone tissue compared to HA alone. Therefore, new research should focus on improving the mechanical properties of HA complexes by adding a reinforcing agent with the same bioactivity, or by adding the smallest possible volume of reinforcing agents without drastically decreasing the bioactivity of HA.

Meanwhile, after researchers reported that carbon nanotubes (CNTs) exhibit bioactive properties, the application of CNTs increased in the biomedical field [8,9]. CNTs were first discovered by lijima in 1991 [10] and have since been studied for various uses, including displays, fuel cells, and medical applications, due to their outstanding thermal, electrical, and mechanical properties resulting from their unique molecular structure [11,12]. A CNT has the shape of a graphite sheet rolled into a cylinder and may exhibit a variety of structures, including single-walled CNTs (SWCNTs), multi-walled CNTs (MWCNTs), and rope nanotubes. Furthermore, CNTs also have a large aspect ratio. CNTs are reported to have a high elastic modulus of 1 TPa or more, and a high tensile strength up to 30 GPa [13,14], although this differs depending on the synthesis method. These excellent mechanical properties have led to many studies on the use of CNTs as a reinforcing agent to produce high strength composite materials [11,15,16]. Other studies report improved mechanical properties in an HA coating layer by adding CNTs as a reinforcing agent due to the biological characteristics and superior mechanical properties of CNTs [17,18]. CNTs are a very interesting material with potential biomedical applications.

MWCNT-HA composites have been steadily studied to use as biomaterials. MWCNT-HA composites produced by various methods can be used for dental and orthopedic biomaterials since it was identified that they have good biocompatibility through various in vivo and in vitro tests [19,20,21]. Further, the studies for MWCNT-HA coating on the surface of implants were actively conducted to enhance the bioactivity of implants. Various methods, such as electrophoretic deposition [18], plasma spraying [22], and sol-gel dip-coating [23] have been developed to coat MWCNT-HA on titanium alloys. Among them, the coating produced by sol-gel dip-coating has a higher binding force (44 MPa) than coatings produced by electrophoretic deposition (2.8 MPa) and plasma spraying (29 MPa). In this study, MWCNTs-HA was coated on titanium using sol-gel dip-coating with excellent binding force to improve the bioactivity, and then their surface characteristics and biocompatibility were evaluated.

Our study used the sol-gel method to produce a MWCNTs-HA nanocomposite with various contents of MWCNTs. The composite was used to coat the surface of pure titanium in order to improve bioactivity. Changes in the surface properties of the coated titanium were examined, and the impact on cell growth from the CNT-HA coated titanium surface was surveyed.

## 2. Materials and Method

### 2.1. Material Preparation

#### 2.1.1. Synthesis

HA was produced using the sol-gel method. Here, 0.5 M calcium nitrate tetrahydrate (Ca(NO_3_)_2_·4H_2_O) (Sigma-Aldrich, Saint Louis, MO, USA) and 0.3 M ammonium phosphate dibasic ((NH_4_)_2_·HPO_4_) (Sigma-Aldrich, Saint Louis, MO, USA) were used as reagents, and the Ca:P ratio was set to 1.67. To produce HA, a 0.3 M ammonium phosphate dibasic solution was added to a 0.5 M calcium nitrate tetrahydrate solution at a 1:1 ratio. Here, an ammonia solution (SHOWA, Urawa, Japan) was added to raise the pH of the solution to at least 10. The solutions were stirred for 3 h. After, the solution aged for 24 h at 25 °C in a Forced Convection Oven (OF-12GW, JEIO TECH, Deajeon, Korea) and was dried to obtain the HA powder.

#### 2.1.2. Nanocomposite Preparation

The MWCNTs (Carbon Nano-material Technology, Incheon, Korea) were stirred for 8 h at 120 °C in 60% nitric acid. The solution was then filtered using secondary distilled water until the pH reached 7, and dried at 55 °C to produce MWCNTs-COOH.

To produce the MWCNTs-HA, 0.1, 0.5, and 1 wt % MWCNTs-COOH were added to a nitrate solution and stirred with ultrasonic waves for 30 min each. Then, a phosphate solution was added one drop at a time. Here, an ammonia solution was added so that the pH reached at least 10. MWCNTs-HA powders were obtained after stirring for 3 h and aging for 24 h. The HA and MWCNTs-HA powders were sintered for 30 min at 500 °C and used accordingly.

#### 2.1.3. The Deposition of the Synthesized Composite on the Ti Surface

Pure titanium (Grade II, HYUNDAI TITANIUM, Incheon, Korea) was polished up to (#600–#1000) using silicon carbide sandpaper. After polishing, the titanium was washed with ethanol and distilled water for 10 min each and subsequently dried. This preparation procedure was intended to improve the cohesion of the MWCNTs (Carbon Nano-material Technology, Incheon, Korea) on the titanium surface. The titanium was placed on a hot plate heated to 120 °C, and 100 μL of 0.25 M 3-aminopropyltriethoxysilane (Sigma-Aldrich, Saint Louis, MO, USA) was dropped on the titanium in 100 μL intervals for silane finishing. The silane finishing process was repeated twice. Next, the specimen was allowed to cool to room temperature, and the sample was placed in distilled water for ultrasonic cleaning.

### 2.2. Surface Treatment

The prepared HA and MWCNTs-HA powders were dispersed in ethanol to produce a suspension. Each of the prepared suspensions (0, 0.1, 0.5, and 1 wt % MWCNTs-HA) were divided into 20 portions of 20 μL each and applied to the surface of the silane-treated titanium. Then, titanium surfaces treated with 0, 0.1, 0.5, and 1 wt % MWCNTs-HA were obtained. The MWCNTs-HA treated titanium specimen was finally heated for 2 h at 450 °C.

### 2.3. Surface Analysis

A transmission electron microscope (TEM) (H-7650, HITACHI, Tokyo, Japan) and Fourier-transform infrared spectroscopy (FT-IR) (Frontier, Perkin Elmer, Foster City, CA, USA) analysis was performed at wavelengths of 400 to 4000 cm^−1^ and was used to assess the interaction between HA and MWCNTs. To survey phase changes of HA and the MWCNTs-HA mixed powder, an X-ray diffractometer (XRD) (X’pert Pro Powder, PNAalytical, Almelo, The Netherland) was used to perform X-ray diffraction analysis from 0° to 50° at 4° per min scan rate. After coating the microstructure with osmium, the MWCNTs-HA surface was examined using a field emission scanning electron microscope (FE-SEM) (SU-70, HITACHI, Tokyo, Japan).

### 2.4. Cell Test (In Vitro Test)

#### 2.4.1. Cell Proliferation Verified through water soluble tetrazolium (WST)

WST-8 (water soluble tetrazolium) assay was performed as follows. Each of the surface-treated specimens were placed on a 24-well plate. TMC3T3-E1 cells (osteoblast of a mouse) were disseminated to 2 × 10^4^ cells mL^−1^ and were left to cultivate on the surface of the specimen for 2 and 5 d. After 2 and 5 d periods, the medium was removed and a mixture of a CCK-8 (Enzo) solution and α-MEM medium was divided into 500 μL portions and stored for 30 min inside a 5% CO_2_ culture medium. After placing it onto a 96-well plate in 200 μL portions, the optical density was measured at 450 nm using an ELISA reader (Molecular devices, EMax, San Jose, CA, USA).

#### 2.4.2. Cell Formation Observation

The osteoblast’s cohesion appearance and form under each condition was observed using an FE-SEM (SU-70, HITACHI, Tokyo, Japan). Each surface-treated specimen was placed on a 24-well plate, and the cell density was disseminated to 2 × 10^4^ cells mL^−1^. The medium was removed after 2 and 5 d, and the cells were washed using phosphate buffered silane (PBS). Then, the specimens were fixed for 2 h at 4 °C using 2.5% glutraraldehyde and 1% osmium tetroxide. Ethanol gradient solutions (50%, 60%, 70%, 80%, 90%, and 100%) were then used to dehydrate each specimen for 15 min. A plasma sputter coater (Emscope SC500K, London, UK) was used to deposit a platinum coating in an argon atmosphere before capturing FE-SEM images.

#### 2.4.3. Alkaline Phosphatase (ALP) Activity

ALP activity was evaluated using a TRACP and ALP assay kit (TakaRa, Shiga, Japan). The MC3T3-E1 cells were cultivated for 7 and 10 d at a cell density of 2 × 10^4^ cells mL^−1^ on top of a 24-well plate coated with powder from each group. The medium was removed, and the plate was washed with a saline solution. Then, 1 mL of a P-nitro-phenyl phosphate (pNPP) solution mixed with an extraction solution and alkaline phosphatase buffer solution was added. The cells were placed inside an incubator at 30 °C for 1 h, then they were placed inside a 96-well plate in 200 μL portions, and the optical density was measured at 405 nm.

#### 2.4.4. Statistical Processing Analysis

Each experiment was performed five times, and all results were statistically processed using a variance method (ANOVA) (*p* > 0.05).

## 3. Results

**Figure 1:**
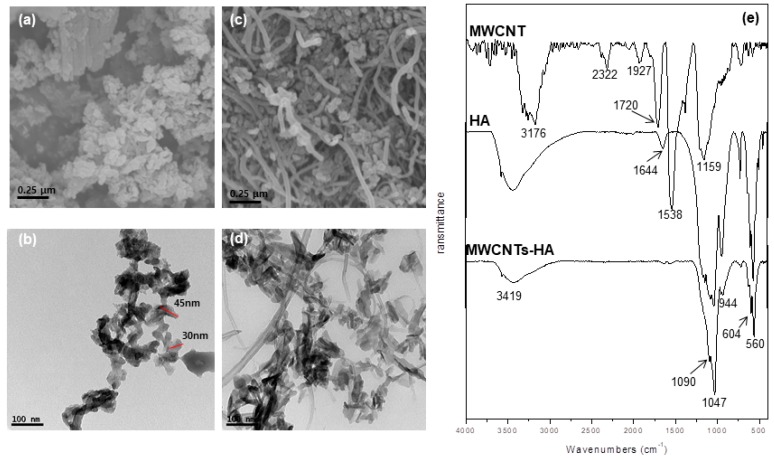
(**a**) Field emission scanning electron microscope (FE-SEM) and (**b**) transmission electron microscopy (TEM) image of sol-gel synthesized hydroxyapatite (HA) powders, (**c**) FE-SEM image and (**d**) TEM image of multi walled carbon nanotubes-hydroxyapatite (MWCNTs-HA) powders, and (**e**) Fourier-transform infrared spectroscopy (FT-IR) spectra of MWCNT, HA and MWCNTs-HA powders.

The microstructure of the sol-gel synthesized HA and MWCNTs-HA nanocomplex powders was observed using FE-SEM and TEM. As shown in Figure 1a,b, the HA powders had a uniform, nano-size rice shape with a diameter of approximately 35 nm and a length of about 45 nm. The MWCNTs-HA nanocomposite particles in Figure 1c,d show that the HA nanoparticles and MWCNTs combined and became dispersed. FT-IR spectra from the MWCNTs, HA, and MWCNTs-HA are shown in Figure 1e. OH stretching bands in MWCNTs were found between 3176 and 2322 cm^−1^, and the C=O, C=C, and C–O bonds were found at 1720, 1538, and 1159 cm^−1^, respectively. P–O bonds from HA were found at 567, 604, 1047, and 1089 cm^−1^, and OH bonds were found at 3419 cm^−1^. The peak from MWCNTs-HA matched that from HA, and a weak C=C bond was found at 1538 cm^−1^.

**Figure 2:**
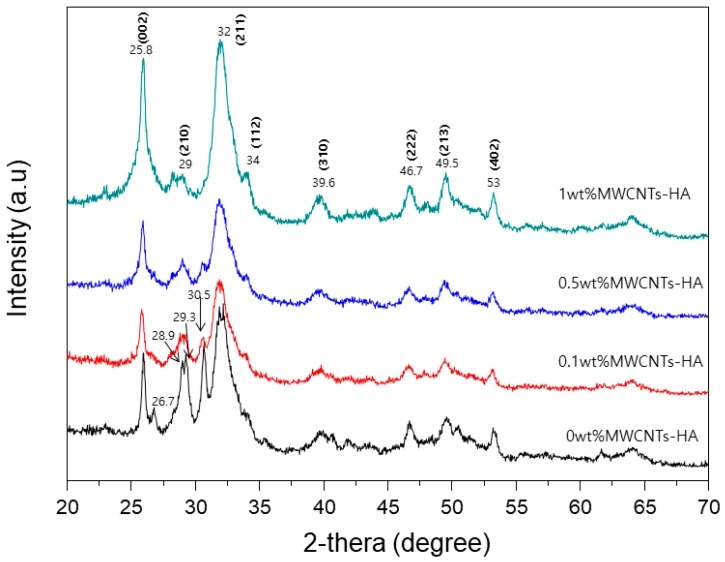
X-ray diffraction (XRD) pattern of MWCNTs-HA composites.

An XRD pattern from the MWCNTs-HA nanocomposite is shown in Figure 2. The 2θ values indicate crystalline HA (JXPDS card # 09-0432). No new peaks were found in the XRD patterns when MWCNTs were added. However, the shape of the peak became sharper as the content of MWCNTs increased. No peaks corresponding to CNTs were observed.

**Figure 3:**
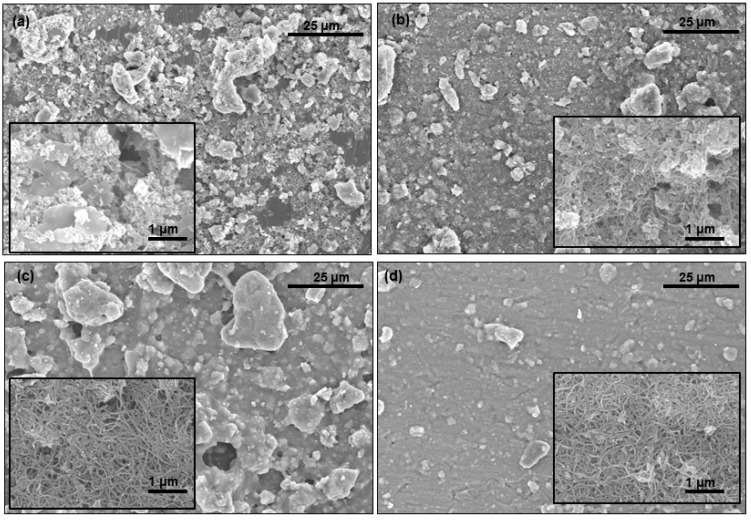
FE-SEM image of the MWCNTs-HA composites with various MWCNT contents (**a**) 0 wt %, (**b**) 0.1 wt %, (**c**) 0.5 wt % and (**d**) 1 wt %.

Figure 3 shows FE-SEM images of the titanium surface coated with MWCNTs-HA. Only the HA-coated group is shown in Figure 3a, and small particles are attached to the rough surface. Figure 3b–d shows the surface treated with increasing MWCNT concentration. No major differences in the HA-coated surface and the MWCNTs-HA coated surface could be seen when the surface was examined at low magnification, and the existence of MWCNTs-HA could not be verified. However, at high magnification, the presence of MWCNTs was verified on the surface of titanium, and it was found that MWCNTs and HA became aggregated. As MWCNTs filled up the spaces between uneven particles on the surface, the 0.1 wt % MWCNTs-HA surface (Figure 3b) was more even than the sample shown in Figure 3a. MWCNTs and HA particles were severely aggregated on the 0.5 wt % MWCNTs-HA surface. The 1 wt % MWCNTs-HA surface was more even than the other surfaces.

**Figure 4:**
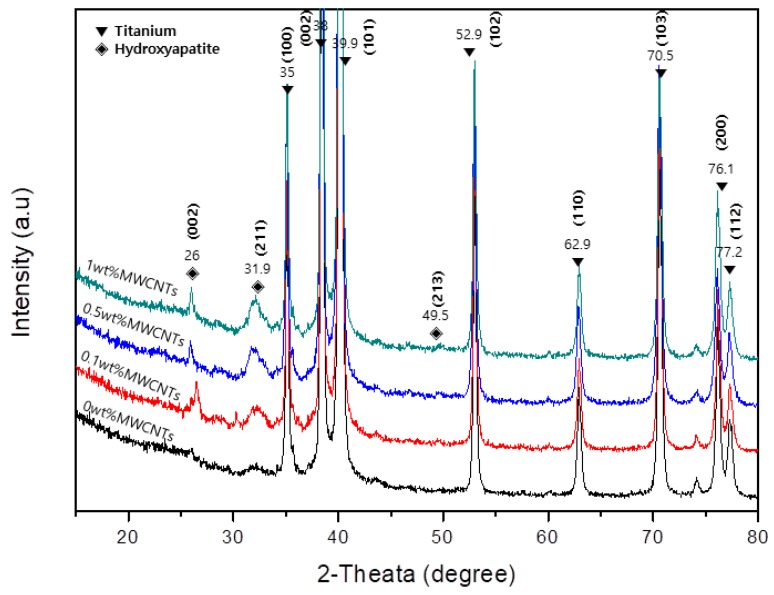
XRD pattern of MWCNTs-HA composites coating on titanium.

Figure 4 shows the XRD pattern of the titanium surface coated with 0, 0.1, 0.5, and 1 wt % MWCNTs-HA. The main titanium peaks are visible in the XRD patterns, and the titanium peak did not change after applying the surface treatment. The HA phase was discovered at 2θ values of 26°, 31.9°, 49.5°, and 52.9°. As the volume of CNTs increased, the HA peak became sharper.

**Figure 5:**
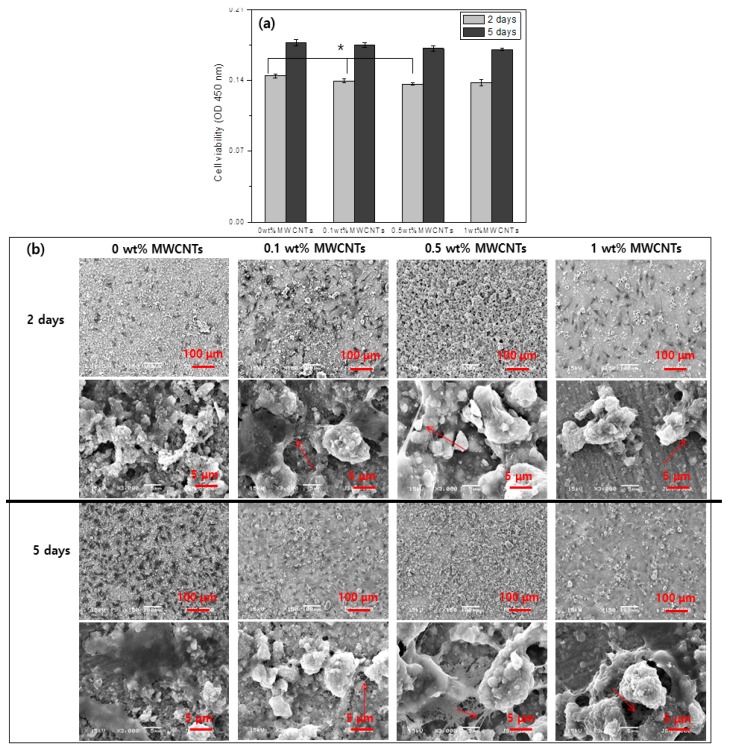
(**a**) The cell viability analysis by water soluble tetrazolium (WST). (**b**) The cytoskeleton analysis of the MC3T3-E1 cells for 2 and 5 days culturing on the MWCNTs-HA nanocomposite coating with various MWCNT contents (0, 0.1, 0.5 and 1 wt %) on titanium.

Figure 5 shows an evaluation of cell proliferation of the surface treated titanium as a function of MWCNT volume. Figure 5a shows the WST assay results after cells were cultivated for 2 and 5 d. Osteoblasts proliferated as cultivation time increased in all groups. After 2 and 5 d of cell cultivation, cell proliferation increased as the volume of MWCNTs decreased, but there were no statistically significant differences between the two groups.

Figure 5b shows the appearance of cells on the surface of each surface-treated group. It was difficult to see the cells at low magnification due to the round surfaces coated with MWCNTs-HA. However, at high magnification, the filopodia of cells in the group coated with 0.1, 0.5, and 1 wt % MWCNTs-HA was less developed than in the uncoated group.

**Figure 6:**
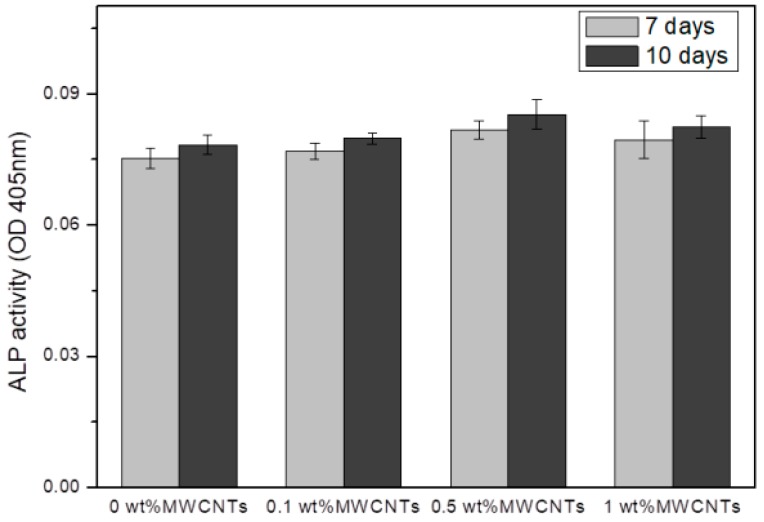
Alkaline phosphatase (ALP) activity of the MC3T3-E1 cells. The cells were cultured for 7 and 10 days on the MWCNTs-HA nanocomposite with various MWCNT contents (0 wt %, 0.1 wt %, 0.5 wt % and 1 wt %) coating on titanium.

Figure 6 shows the ALP activity of the titanium surface coated with MWCNTs-HA after cell cultivation for 7 and 10 d. The results on the 7th and 10th days of cell cultivation showed that ALP activity increased as MWCNT concentration increased when the content of MWCNTs was 0.5% or lower, and ALPT activity decreased when the content of MWCNTs exceeded 0.5%.

## 4. Discussion

Extensive research is being conducted on ceramic-CNT composites, especially regarding the various effects of CNTs on the mechanical properties of ceramic-CNT composites [24]. A recent study used HA complexes reinforced with CNTs as materials for bone substitution [25]. Bone and enamel minerals are related to the formation of HA in the synthesis system of a biomimetic environment. Bone and enamel may share HA with the same mineral composition, but they differ in shape and organic matter [26]. Therefore, HA formation is important from a therapeutic approach for tissue treatment. CNTs have a structure that can replace collagen fibers and play the role of a structural stiffener for use as tissue scaffolding. Moreover, the strong electrical and chemical characteristics of CNTs offer an opportunity to monitor implant interaction with matrix components aside from cells by delivering influences derived from tissues, such as electrical stimuli, growth factors, and dielectric substances that participate in osteoblast growth [27]. Composites of CNTs and HA can be developed as a coating, nanocomplex, or hybrid powder for potential use in soft tissue reconstruction [20]. Furthermore, CNT coatings and complexes have been successfully used as substrates for biomineralization and osteoblast growth, proliferation, and normal function [17].

This study used the sol-gel method to produce MWCNTs-HA nanocomposites at various concentrations (0.1, 0.5 and 1 wt % MWCNTs), and a pure titanium surface was coated in order to improve bioactivity. The properties of the MWCNTs-HA coated titanium surface and the impact of the MWCNTs-HA coated titanium surface on cell growth were surveyed. To create complexes mixed with CNTs (which has ~10^3^ aspect ratio) as stiffeners, it is extremely important to disperse the CNTs as evenly as possible in the complex. To this end, this study generated and used a COOH functional group on the surface of the MWCNTs by treating all MWCNTs with acid. The sol-gel method was used to produce a complex MWCNTs-HA powder. The shape of the MWCNTs-HA was verified using SEM and TEM. The results showed that HA with nanoparticles was attached to the surface of long tube-shaped MWCNTs (Figure 1a–d). The OH stretching bands of MWCNTs were observed between 3176 cm^−1^, and the C=O, C=C, and C–O bonds were observed at 1720, 1538, and 1159 cm^−1^, respectively [28]. P–O bonds were found at 567, 604, 1047, and 1089 cm^−1^ from HA, and OH bonds were found at 3419 cm^−1^ [17]. No new peaks were found in the MWCNTs-HA, but in the MWCNTs-HA graph, the peaks at 1047 and 1089 cm^−1^ were sharper than in the HA graph. According to Kaya et al., the separated bands at 1047 and 1089 cm^−1^ were advantageous for forming a crystallized apatite structure [18]. In the XRD pattern of the MWCNTs-HA nanocomposite, crystalline HA was discovered at 2θ values of 23°, 26°, 32°, and 49° (JCPDS card # 09-0432). However, no MWCNT peaks were found in the XRD. The carbon phase could not be verified because the CNT content was too low and carbon peaks with a 2θ = 26° were identical. Moreover, the peak in the graph became sharper as the CNT content increased. In addition, the XRD pattern from the titanium implant surface coated with MWCNTs-HA nanocomposites (Figure 4) did not show a change in titanium’s chemical components with surface treatment, and the HA peak became sharper as more CNTs were added. This implies that there was an increase in the concentration of HA crystals. Due to the negative charge caused by the functional group on the surface of the CNTs, the CNTs were assumed to serve as a favorable location for forming the nucleus of HA [29]. When the CNT surface was well-fitted with an ionic group, calcium or phosphate ions were induced on the surface, and ions with opposite charge were induced to form the nucleus [30]. This nucleus generation was accompanied by crystallization and further crystal growth. The chemical functionalization of CNTs permitted increased hydrophilicity and modified many groups on the surface of the reformed tube, thereby providing a site for the generation of countless nuclei [30]. As a result, fine HA crystals can deposit quickly at high density along the tube axis. As such, if the formation of HA on the CNT surface increased, it contributes to bonding with the surrounding bone tissue and new bone formation.

When the surface of titanium is coated with MWCNTs-HA nanocomposite particles, MWCNTs and HA particles become aggregated on the microstructure of the surface (Figure 3). The particles on the 1 wt % MWCNTs-HA surface were less aggregated than on the 0.5 wt % MWCNTs surface. The 1 wt % MWCNTs-HA surface was more even than the other surfaces. According to previous studies, particles increase in size as the CNT content increases, so long as CNT content was 0.5 wt % or less during formation of HA crystals. However, the particles became smaller once the content exceeded 5 wt % [31]. This study also found that particles became larger on the 0.5 wt % MWCNTs surface, but become smaller on the 1 wt % MWCNTs surface. The surface became wider as the particles increased in size, thereby providing a better environment for cells to attach.

The superior properties of CNTs, such as their ultra-high surface area, electrical conductivity, and mechanical strength, make CNTs an attractive nanomaterial for various applications, including in the biomedical field [32]. An evaluation of the bioactivity and toxicity of composites is necessary for the use of CNTs as a biomaterial. Various research results have found that CNTs present in biocomposites have no harmful effects and improve bioactivity. George et al. [33] studied human epithelial cells and osteoblasts, as well as their reaction with MWCNTs. This study showed that the size and spacing of CNTs are key determinants of a cell’s spread and growth. Price et al. [34] showed that the adhesion of osteoblasts accelerated when carbon nanofibers with a diameter of 60 to 200 nm are generated by chemical vapor deposition through bonds with themselves or with polyurethane. This study verified the proliferation of osteoblasts after cultivation on the surface of titanium coated with MWCNTs-HA at various concentrations for 2 and 5 d (Figure 5). In all groups, osteoblast proliferation increased as cultivation time increased. After 2 d of cell cultivation, cell proliferation increased as the volume of MWCNTs decreased, but there were no statistically significant differences for the 1 wt % MWCNT group. After 5 d of cell cultivation, there were no statistically significant differences between groups with regards to cell proliferation. This shows that MWCNTs do not impact the proliferation of cells. Moreover, upon observing the shape of the cells on the surface of each surface treated group, the filopodia of cells in the group coated with 0.1, 0.5, and 1 wt % MWCNTs-HA were less developed than in the uncoated group. Zanello et al. [9] compared bone formation and proliferation of cells in functionalized and non-functionalized CNTs. They found that the growth of rat osteosarcoma ROS 17/2.8 cells occurred on the surface of as-prepared SWNTs and neutrally-charged polyethylene glycol-functionalized SWNTs. Furthermore, they also claimed that multi-layered CNTs or MWCNTs can be used to control the shape of the cells and cytodifferentiation.

Long-term research is required to determine the survival rate of osteoblasts. Osteoblast differentiation markers that appear during osteoblast cultivation occur based on the degree of cell proliferation and differentiation, and bone is formed through three processes that involve a growth phase, substrate maturation period, and calcification. Of all bone markers, ALP is a marker that appears at the beginning of osteoblast differentiation. It peaks during the substrate maturation period and decreases during the calcification stage. ALP present in bone tissue hydrolyzes phosphate esters to control the metabolism of phosphoric acid and maintain phosphoryl metabolites at a certain level. ALP also plays the role of phosphoprotein phosphatase. In the results of this study, Figure 6 shows the ALP activity on the titanium surface coated with MWCNTs-HA after cell cultivation for 7 and 10 d. The results on the 7th and 10th day of cell cultivation show that ALP activity increased as MWCNTs increased when the content of MWCNTs was 0.5% or lower, and ALPT activity decreased when the content of MWCNTs exceeded 0.5%. When CNTs were included, cell differentiation was found to increase. According to previous studies, the surface of CNTs was suitable for apatite mineralization. CNTs accelerate new bone formation and bonding with coated implants [35].

Therefore, HA crystals form more easily on the surface of titanium coated with MWCNTs-HA nanocomplex particles as the amount of CNTs increases, and 0.5 wt % MWCNTs-HA particles increase in size. The cell proliferation results and cytodifferentiation of MWCNTs-HA composites showed that cell proliferation was successful regardless of the MWCNT concentration, and cytodifferentiation was highest on the 0.5 wt % MWCNTs-HA surface. MWCNTs-HA composites can be used as a bone substitute.

## 5. Conclusions

In this study, sol-gel processing method was used to produce MWCNTs-HA nanocomposites with different MWCNTs concentration. The nanocomposites were used to coat the surface of titanium such that MWCNTs could be used as bone substitutes. The structural characteristics of a MWCNTs-HA complex powder showed that HA nanoparticles bonded to the surface of the MWCNTs. Crystallization on the MWCNTs-HA nanocomposite showed that crystallization of HA was improved as the concentration of MWCNTs increased.

The cell test results with different MWCNT concentrations showed that cell proliferation increased regardless of the MWCNT concentration, and the filopodia of cells developed in the specimen that included MWCNTs. Moreover, cytodifferentiation was highest on the 0.5 wt % MWCNTs-HA surface.
